# Graphene-based nonvolatile terahertz switch with asymmetric electrodes

**DOI:** 10.1038/s41598-018-20047-3

**Published:** 2018-01-24

**Authors:** Yan Li, Hui Yu, Xinyu Qiu, Tingge Dai, Jianfei Jiang, Gencheng Wang, Qiang Zhang, Yali Qin, Jianyi Yang, Xiaoqing Jiang

**Affiliations:** 1 College of Information Science and Electronics Engineering, Zhejiang University, Hangzhou, 310027 China; 2 College of Information Engineering, Zhejiang University of Technology, Hangzhou, 310014 China

## Abstract

We propose a nonvolatile terahertz (THz) switch which is able to perform the switching with transient stimulus. The device utilizes graphene as its floating-gate layer, which changes the transmissivity of THz signal by trapping the tunneling charges. The conventional top-down electrode configuration is replaced by a left-right electrode configuration, so THz signals could transmit through this device with the transmissivity being controlled by voltage pulses. The two electrodes are made of metals with different work functions. The resultant asymmetrical energy band structure ensures that both electrical programming and erasing are viable. With the aid of localized surface plasmon resonances in graphene ribbon arrays, the modulation depth is 89% provided that the Femi level of graphene is tuned between 0 and 0.2 eV by proper voltage pulses.

## Introduction

The terahertz technology has gained increased research attention due to its industrial and academic applications^1,2^. Various approaches, including those based on active metamaterials^3–5^ and two-dimensional materials^6–8^, have been investigated over the past decade to manipulate the propagation of electromagnetic waves from RF to THz frequencies. However, most of them are volatile devices, as they return to original states once external stimuluses are removed. Nonvolatile photonic devices, which can sustain the transformed properties with short triggering stimuluses rather than persistent signals, hold an apparent advantage of saving the energy in the advent of photonic technologies. More importantly, the persistent photonic memory effect of these devices lend themselves to a variety of applications including protective optical/terahertz circuitry, adaptable transformation-optics devices, dynamically reconfigurable optical/THz networks or metamaterials, electrically controllable photonic memory^9–15^, and so on. Furthermore, most planar lightwave circuits, especially those built on the silicon-on-insulator substrate that have high refractive index contrasts and strong optical confinements, are sensitive to fabrication tolerance. Therefore, many properties of practical photonic circuits such as the resonance wavelength would inevitably drift from their design values. Nonvolatile devices then can be incorporated into these photonics circuits as a post-trimming technique to compensate permanent errors.

**Figure 1:**
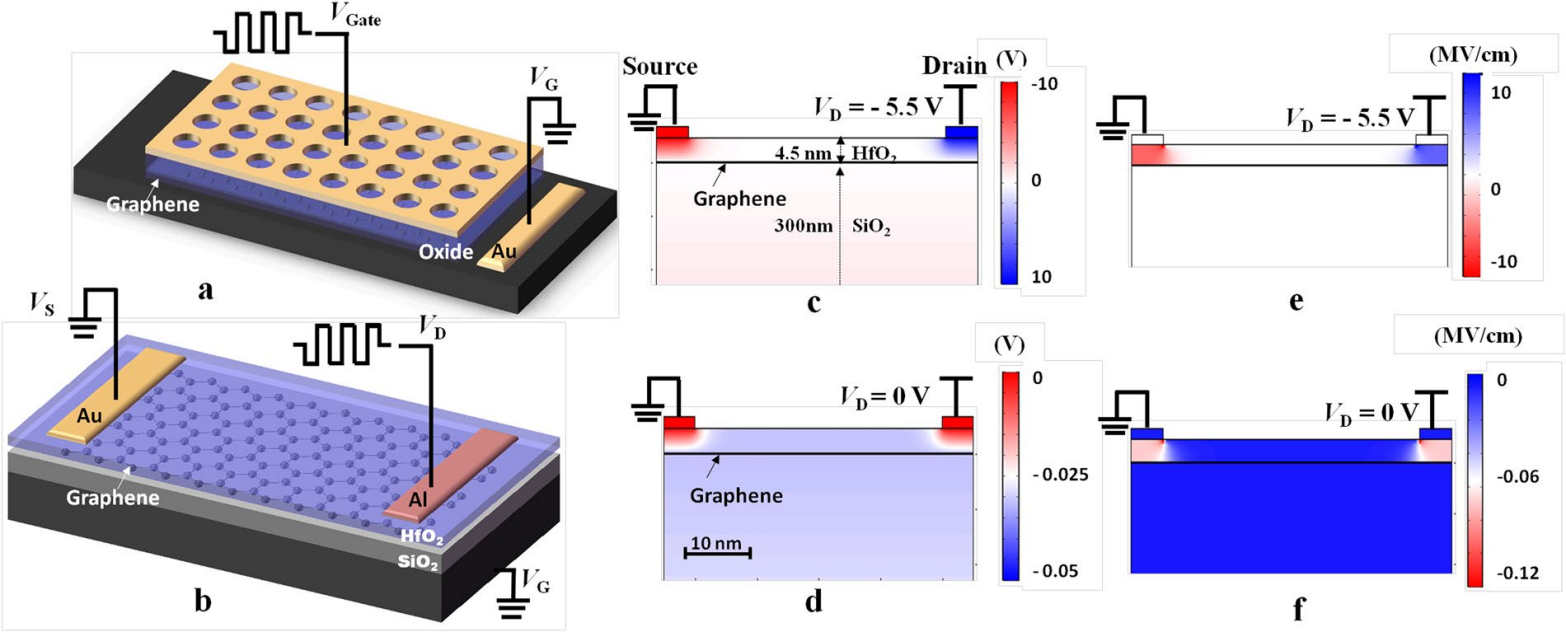
Device structures and numerical simulation results of electrostatic potential distributions. 3-D schematic diagrams of (**a**) the traditional FG memory, and (**b**) the graphene-based nonvolatile THz switch. The potential distributions in the nonvolatile THz switch at (**c**) the programming mode and (**d**) the retention mode, and the corresponding distributions of vertical electrical fields (**e**,**f**).

Although a lot of nonvolatile devices, e.g., those based on floating gate structures^14–16^, phase-change materials^11–13^, organic materials^17^, ferromagnetic materials^18^, have been well developed for data storage, few of them turn out to be a feasible solution to implement the nonvolatile THz switching. The reason is that most of those devices contain functional materials or top-gate electrodes that are nontransparent at THz frequencies. For example, the floating-gate (FG) shown in Fig. 1(a) is so far the most successful configuration for the flash memory. It relies on charges stored in the FG layer to tune the threshold voltage of the transistor. In^19,20^ graphene is employed as the FG layer to store charges, which exhibits improved performance in terms of endurance, writing speed and operation voltage. However, THz signals cannot propagate through this configuration, since it demands a metallic control gate on the top, which is highly lossy at THz frequencies. Although metamaterial structures^5,9–12^, for example, a periodic hole array patterned on an Au film as shown in Fig. 1(a), can be utilized to enhance the transmission through the metal layer, the limited bandwidth and the additional loss are tricky issues. Generally speaking, to design a technically viable and high-performance nonvolatile THz device still remains a challenge.

In this paper, we theoretically propose a nonvolatile THz switch by employing the graphene as the floating gate layer. The extraordinary optoelectronic property of graphene allows stored carriers to significantly tune the transmissivity of the THz signal. The conventional control-gate layer that covers the full device is replaced by source and drain electrodes made of different metals at the two sides as shown in Fig. 1(b). Hence the space between two electrodes acts as an open window for the THz transmission. With an appropriate thickness of tunneling layer (HfO_2_), electrons from the drain electrode can tunnel through the HfO_2_ layer and be stored in the FG (graphene) layer under a negative drain voltage. Meanwhile, since the tunneling barrier under the source electrode between graphene and HfO_2_ is higher than that between the drain electrode and HfO_2_, electrons that tunnel out to the source electrode from the FG can be neglected. Therefore, electrons can accumulate in the FG. Similarly, under a positive drain voltage, the electrons stored in the FG can tunnel back to the drain, while electrons that tunnel into the FG from the source is negligible. Electrons therefore can be erased from the FG also due to the unequal tunneling barrier heights at the two electrode terminals. The nonvolatile switching is finally realized by taking advantage of different tunneling barriers at the two terminals.

Although the proof-of-principle design of the THz nonvolatile switch here is based on free-space optics with the THz wave normally incident on the graphene layer, this scheme is also well adapted to various waveguide-based switches operating at THz and optical wavelengths. A lot of graphene-based photonic modulators have been reported on either dielectric^7,21,22^ or plasmonic waveguides^23–25^. We note that active graphene layers in many of these modulators can be readily replaced by the graphene-based floating-gate in Fig. 1(b) with the two asymmetrical electrodes locating at the two sides of the waveguides. Since waveguide modes are confined to propagate along the graphene plane, the interaction length between THz/optical waves and graphene is extended, and hence Performance metrics in terms of power consumption, footprint, and modulation depth can be improved significantly for nonvolatile switching.

## Results

### Graphenen-based nonvolatile switching structure

A schematic diagram of the nonvolatile switch is shown in Fig. 1(b), which is built on the grounded silicon substrate. Monolayer graphene (work function: *ψ*_g_ = 4.6 eV^19^) vertically stacking on a 300-nm-thick SiO_2_ layer works as the floating-gate layer. Another 4.5-nm-thick HfO_2_ layer (*ψ*_HfO2_ = 2.5 eV^26^) acts as the tunneling/blocking layer. The drain and source electrodes on top are made of aluminum (*ψ*_Al_ = 4.08 eV) and gold (*ψ*_Au_ = 5.1 eV^27^), respectively. To reveal the operation principle, the finite elements methods (Comsol Multiphysics) is utilized to model the electrostatic potential distribution in this device. The distance between the two electrodes is set to be 50 nm to better display the vertical configuration. It should be noted that the width of this transmission window for THz beams is on the scale of centimeters in practical devices. However, the electrostatic potential distribution in practical devices is similar to that presented in Fig. 1(c) and (d). On the other hand, although there are non-uniform fringe electrical fields at edges of electrodes as shown in Fig. 1(e) and (f), it is reasonable to assume fields between two electrodes and graphene are uniform when we calculate the tunnelling current. The reason is that widths of practical electrodes are on the scale of millimeter. For most areas below electrodes that are away from edges, electrical fields are uniform. In programming and erasing modes, large potential differences and accordingly high electrical fields are constructed between drain/source and graphene to enhance the tunneling of electrons; while low electrical fields are induced by charges stored in the FG layer for the retention mode. The extremely high conductivity of the graphene makes it an equipotential surface, which enables the electrons tunneling from the drain to spread throughout the entire graphene layer^20^. Therefore, the potential difference from drain to graphene almost equals to that from graphene to source as shown in Fig. 1(c) and (d). Consequently, different metals can be utilized to induce imbalanced tunneling currents, which is crucial to implement necessary program/erase operations of a nonvolatile device as will be explained below.

### Physical mechanism of the nonvolatile operation

**Figure 2:**
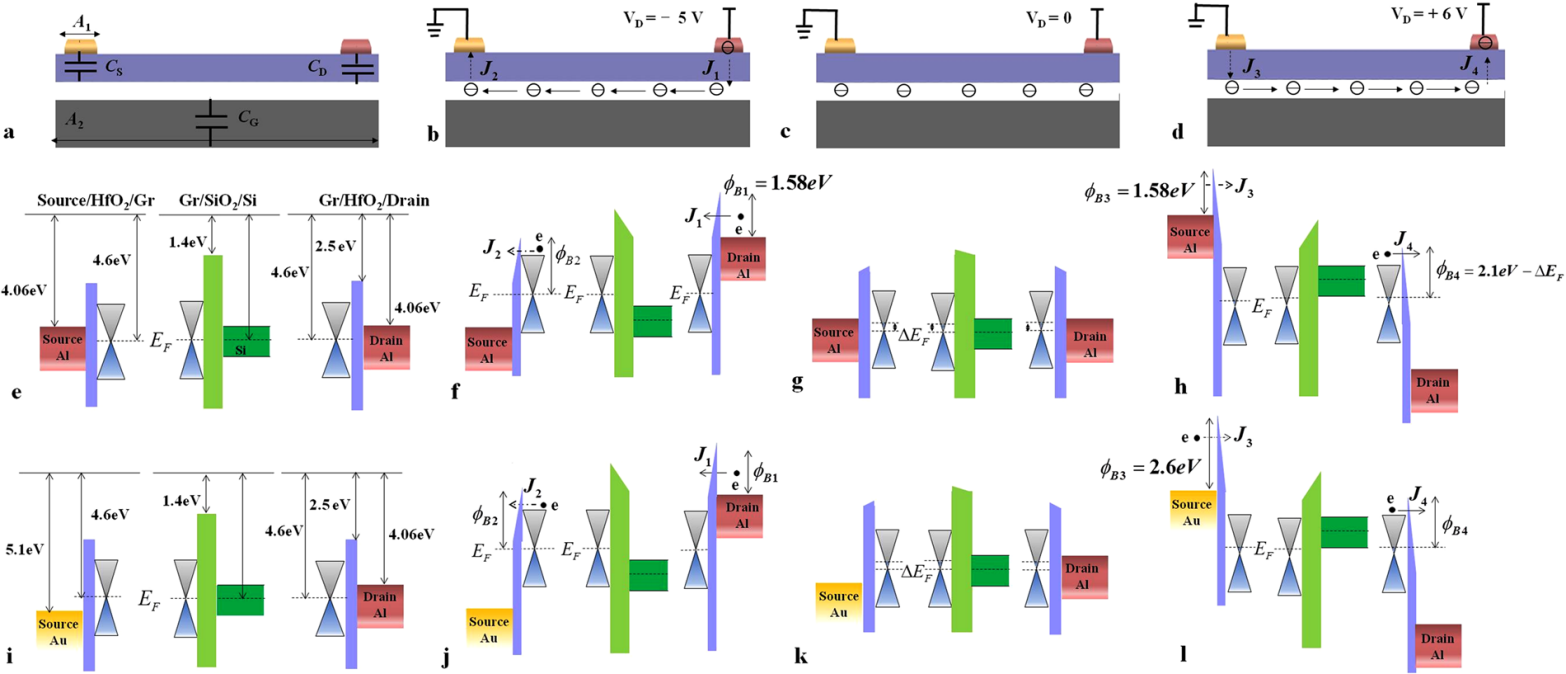
Different operation modes and corresponding vertically band diagrams of the nonvolatile THz device. (**a**) Initial mode (before metal/oxide/graphene contact) (**b**) programming mode, (**c**) retention mode and (**d**) erasing mode; the corresponding energy band diagrams for the symmetric electrodes (**e**–**h**) and asymmetric electrodes (**i**–**l**). The dashed line arrows indicate the tunneling directions of electrons between two electrodes and graphene; while the solid ones indicate the spreading of electrons in the graphene layer.

To explain the necessity of using different metals for source and drain, we at first examine the case of both electrodes being made of the same metal, for example, Aluminum (Al). It is known that in a graphene MOS structure, charges will accumulate in graphene provided that work functions of graphene and metal are different. This localized metal-induced doping effect leads to internal built-in electrical fields inside the graphene, and hence bend its energy band. However, these fields only exist in narrow regions (~0.2 μm) adjacent to drain/graphene and source/graphene interfaces^28^, which is far less than the distance between the two terminals of our device. Therefore, the band bending is neglected to simplify the band diagrams. Resultant directions of tunneling currents and band diagrams at different operation states are shown in Fig. 2. Figure 2(e) depicts the initial energy band (before contact) without any external voltage signal applied on the device. In Fig. 2(b), a negative programming signal (*V*_D_ < 0) is applied on the drain. As already analyzed in Fig. 1, nearly one half of the voltage drops between drain and graphene, while the other half drops between graphene and source. The corresponding band diagram in Fig. 2(f) indicates that the barrier height between drain and HfO_2_ (*ϕ*_B1_ = *ψ*_Al_ − *ψ*_HfO2_ = 1.58 eV) is lower than that between graphene and HfO_2_ (*ϕ*_B2_ = *ψ*_g_ − *ψ*_HfO2_ = 2.1 eV). Since the tunneling probability strongly depends on the barrier height, the tunneling current cross the right barrier is greater than that cross the left barrier (*J*_1_ ≫ *J*_2_), and then electrons accumulate in the FG layer. In the retention mode (Fig. 2c), the voltage applied on the drain is removed. Due to the aggregated electrons (*Q*_FG_ ~3 × 10^12^/cm^2^) in the FG layer, potential and Femi level of graphene rise by ~0.08 V and ~0.2 eV, respectively. Tunneling currents from the FG layer to the source and drain is negligible as a result of the weak electrical field in the blocking layer. Therefore, electrons could be stored in the FG layer for a long period before completely leaking out. In the erasing mode of Fig. 2(d), a positive voltage is applied on the drain which is supposed to clear electrons in the FG layer. As the work function of charged graphene layer (4.4 eV) still outnumbers that of the Al (4.06 eV), the tunneling current from source to the graphene is greater than that from the graphene to the drain, i.e., *J*_3_ ≫ *J*_4_ as shown in Fig. 2(h). There is a net electron tunneling current to increase the electron density in the FG layer. Therefore, the device is unable to accomplish the electrical erasing. In fact, as long as the device employs the same metal for source and drain, either electrical programming or erasing is forbidden.

**Figure 3:**
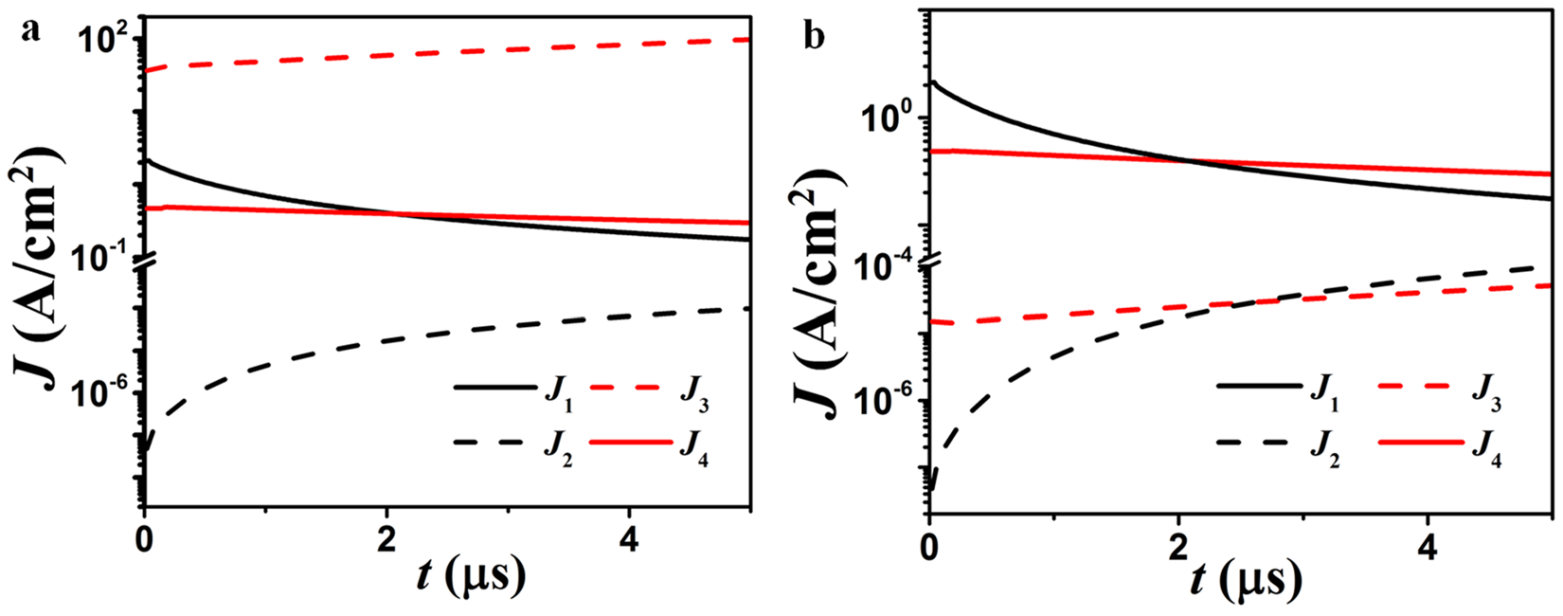
The time responses of the tunneling current density for different electrodes. (**a**) Symmetric Aluminum/Aluminum and (**b**) asymmetric Aluminum/Gold electrodes as drain/source.

To solve this issue, the Al source is replaced by a Gold (Au) source. The different work functions of Al and Au lead to an asymmetrical energy band structure as shown in Fig. 2(i). For the programming and retention modes presented in Figs 2(j) and 3(k), accumulation and leaking of electrons are analogical to those depicted in Fig. 2(f) and (g). However, since the work function of charged graphene (4.6 ± 0.2 eV) is less than gold (5.1 eV), the barrier height on the left side is higher than that on the right side during the erasing process (*ϕ*_B3_ > *ϕ*_B4_) as shown in Fig. 2(l). The tunneling current *J*_3_ is accordingly insignificant as compared with *J*_4_. Therefore, the stored electrons could be erased by a positive voltage due to the asymmetric-electrode configuration.

A quantitative calculation is provided below. Considering the two-dimensional character and band structure peculiarities of graphene, a rigorous treatment should resort to the Bardeen transfer Hamiltonian approach, as it has been utilized to explain tunneling currents in relevant two-dimensional systems^29,30^. However, a strict calculation based on this approach is much more cumbersome than that based on the Fowler-Nordheim (FN) theory^31^. The FN theory is initially built on the free-electron theory of metals and leads to a very simple relationship between the density of emission current and the applied electric field. Strictly, the FN model applies only to bulk systems, e.g., metal/insulator/crystalline solid. However, FN equations are also frequently used as an approximation to describe low dimensional systems, including nanocrystals^32,33^ and two-dimension materials^34,35^. For example, the FN model captures measured tunneling currents in a graphene/insulator/metal system quite well with proper modifying factors in ref.^35^. Based on these considerations, we employ the FN theory without considering the linear energy-momentum relation of graphene. This greatly simplifies the calculation with acceptable accuracy. Regarding the applicability of the FN model to the graphene/insulator/metal interfaces in our device, we discuss how the density of states of graphene affects the tunneling current density in the Supplementary Information. However, a rigorous treatment of the tunneling current is still suggested for our future work.

The conventional FN formula of tunneling current describe the field-assisted emission of electrons under a very high electrical field (*E*_D_ > *qϕ*_B1_/*d*). It can be used to calculate tunneling currents in programming/erasing modes, but is not suitable for the retention mode (*V*_D_ ≈ 0) of very weak electrical field. Generalized formulas are derived by Simmons which enable the calculation of tunneling currents for several different voltage ranges^36^. According to Simmons’ formulas, tunneling current densities *J* at programming/erasing and retention modes can be calculated as

1 $$\begin{array}{rcl}J & = & {A}_{1}{\varphi }_{{\rm{B}}}^{1/2}\exp (-{C}_{1}{\varphi }_{{\rm{B}}}^{1/2}),\,({{\rm{V}}}_{{\rm{D}}}\approx 0)\\ J & = & {A}_{2}{E}^{2}\exp (-{C}_{2}/E).\,({E}_{{\rm{D}}} > q{\varphi }_{{\rm{B1}}}/d)\end{array}$$

definitions of coefficients *A* and *C* are: *A*_1_ = *q*^2^(2 m^*^)^1/2^/(4*d π*^2^ *ℏ*^2^) and *C*_1_ = 2*d*(2 m^*^)^1/2^/ $\hslash$, *A*_2_ = *q*^3^/(16*π*^2^*ħϕ*_B_) and *C*_2_ = 4(2 m^*^)^1/2^(*ϕ*_B_)^3/2^/3*ħq*, here m* denotes the effective mass of the electron, $\hslash$ is the reduced Plank constant, *ϕ*_B_ is the barrier height and *q* is the electron charge. The electrical field between source and FG (*E*_D_) and that between drain and FG (*E*_S_) are determined by the potential of the floating gate layer *V*_FG_, which depends on the amount of stored electrons *Q*_FG_

2 $${V}_{FG}=\frac{{Q}_{FG}}{{C}_{T}}+\frac{{C}_{S}}{{C}_{T}}{V}_{S}+\frac{{C}_{D}}{{C}_{T}}{V}_{D}+\frac{{C}_{G}}{{C}_{T}}{V}_{G},$$

where *V*_S_, *V*_D_, and *V*_G_ are the potentials of source, drain and ground, respectively. As shown in Fig. 2(a), *C*_S_, *C*_D_, and *C*_G_ represent the capacitances (per unit area) between graphene/source, graphene/drain, and graphene/ground, respectively; and *C*_T_ is their sum (*C*_T_ = *C*_S_ + *C*_D_ + *C*_G_)^37^.

According to the MOS theory, *C*_S_, *C*_D_, and *C*_G_ cannot be simply described by their corresponding oxide capacitances *C*_ox_ alone due to the finite density of states (DOS) of graphene. A part of the applied voltage drops in the graphene layer itself, and this effect can be captured in the quantum capacitance *C*_q_ of graphene, which can be calculated as^38^

3 $${C}_{q}=\frac{2{q}^{2}{k}_{B}T}{\pi {(\hslash {\nu }_{F})}^{2}}In[2(1+cosh\frac{{E}_{F}}{{k}_{B}T})]$$

When the Fermi level of graphene locates at the Dirac point, i.e., *E*_F_ = 0, the quantum capacitance reaches its minimum value of ${C}_{q}({E}_{F}=0)=\frac{2{q}^{2}{k}_{B}T}{\pi {(\hslash {\nu }_{F})}^{2}}In(4)\approx 0.8\,\mu F/c{m}^{2}.$ In contrast, the capacitance of the 300-nm-thick bottom silica layer is *C*_Gox_ = 11.5 nF/cm^2^, which is far less than the quantum capacitance. Hence, the influence of the quantum capacitance on *C*_G_ can be neglected. On the other hand, as the thickness of the HfO_2_ layer is very thin, its capacitance is comparable with the quantum capacitance. Therefore, both *C*_S_ and *C*_D_ should be considered as series connections of their respective oxide capacitances and quantum capacitances: 1/*C*_S_ = 1/*C*_Sox_ + 1/*C*_Sq_ and 1/*C*_D_ = 1/*C*_Dox_ + 1/*C*_Dq_.

The stored-charge density in the FG layer is related to the tunneling currents across the two barriers. It can be calculated as

4 $$\begin{array}{rcl}\frac{{\rm{d}}{Q}_{FG}}{{\rm{d}}t} & = & \frac{{W}_{1}}{{W}_{2}}({{J}}_{1}-{{J}}_{2})\,({\rm{Program}}\,{\rm{mode}}),\\ \frac{{{\rm{dQ}}}_{{FG}}}{{\rm{d}}{t}} & = & \frac{{{W}}_{1}}{{{W}}_{2}}({{J}}_{4}-{{J}}_{3})\,({\rm{Erase}}\,{\rm{mode}}),\end{array}$$

*W*_1_/*W*_2_ is the width ratio of the electrode and the FG layer as shown in Fig. 2. The effect of *V*_G_ can be neglected due to the fact that *C*_G_ is far less than *C*_S_ and *C*_D_.

The discussion above has not considered the influences of different work functions between metal and graphene, and the resultant inevitable doping of graphene^39–41^. If these effects need to be reflected, *V*_D_ should be replaced by corresponding effective voltages: *V*_D_eff_ = *V*_D_ − *V*_D0_. Here *V*_D0_ is drain voltage at the Dirac point without external voltages, whose role is very similar to that of the flat-band voltages in conventional MOS transistors. Hence electric fields *E*_D_ and *E*_S_ can be approximately written as

5 $$\begin{array}{rcl}{E}_{D} & = & \frac{{V}_{{D}_{eff}}({C}_{T}-{C}_{D})}{{C}_{T}d}-\frac{{Q}_{FG}}{{C}_{T}},\\ {E}_{D} & = & \frac{{V}_{{D}_{eff}}{C}_{D}}{{C}_{T}d}+\frac{{Q}_{FG}}{{C}_{T}}.\end{array}$$

here*, ε*_0_, *ε*_r_ are the vacuum permittivity and relative permittivity of HfO_2_, respectively, *d* is the thickness of the tunneling layer.

With the aid of equations (1–5), we can calculate time response of current density in programming (*J*_1_, *J*_2_) and erasing (*J*_3_, *J*_4_) modes. Parameters in the calculation are: *ε*_r_ = 17^42^, *W*_1_ = 1 mm, *W*_2_ = 1 cm, *d* = 4.5 nm, *V*_D_ = −5.5 V and +6 V in programming and erasing modes. In Fig. 3(a), both source and drain are made of aluminum. As already explained, electrons can tunnel to graphene with negligible leakage to source (*J*_1_ ≫ *J*_2_) in the programming mode. However, in the erasing mode the electron density in the FG layer keeps on growing as *J*_4_ ≪ *J*_3_, and therefore they cannot be erased. In Fig. 3(b), Al and Au are used as drain and source electrodes, respectively. Because of their different work functions, the electron transportation between source and graphene is inappreciable in both programming and erasing modes. By reversing the polarity of the drain voltage, we can either increase or decrease the amount of electrons stored in FG layer for different modes.

**Figure 4:**
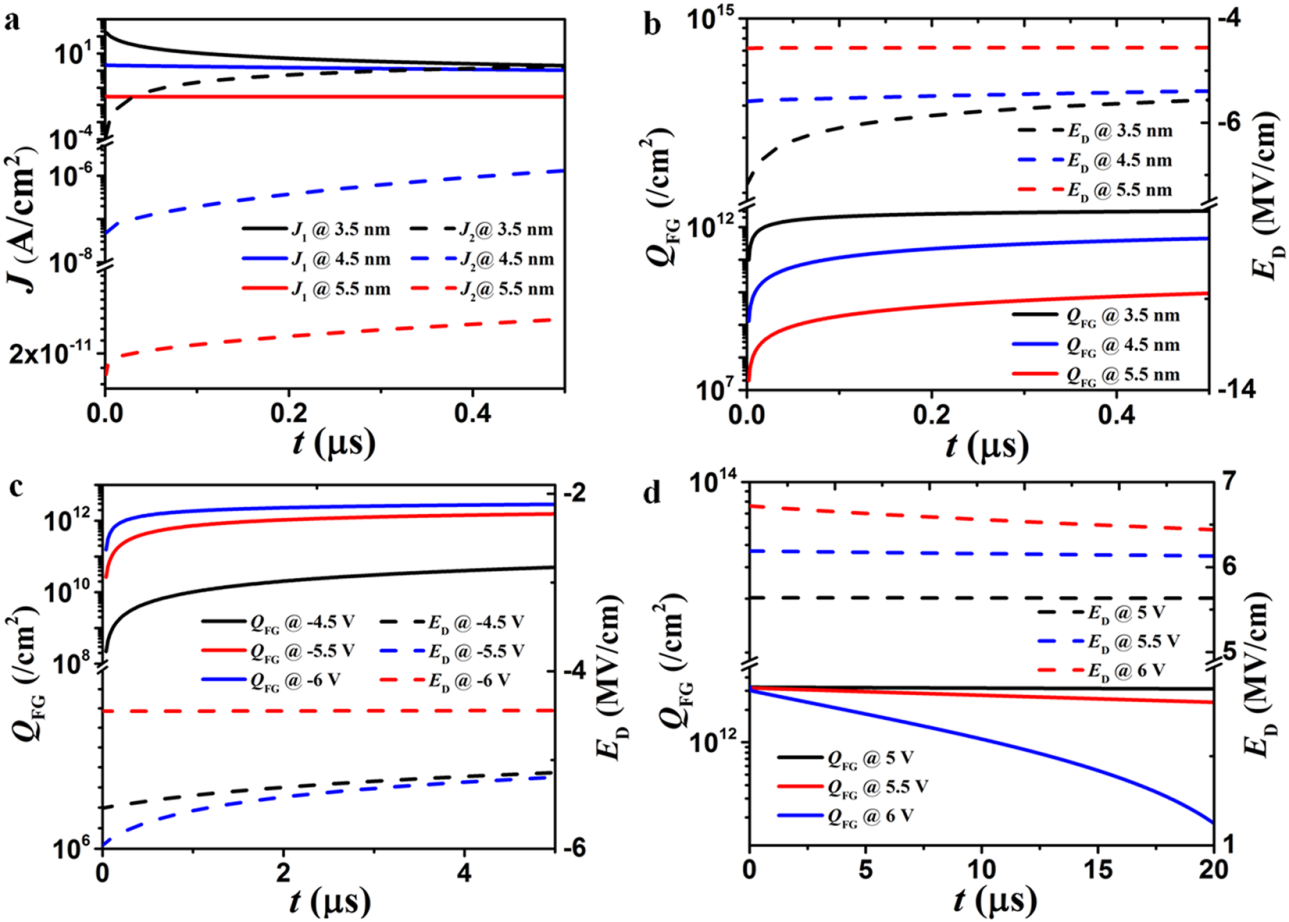
The processes of programming and erasing charges. The time responses of (**a**) tunneling current densities *J*_1_ and *J*_2_, (**b**) electrical field strength between drain and graphene *E*_D,_ density of stored charge *Q*_FG_ for different thicknesses of tunneling layer *d*; the time responses of *Q*_FG_ and *E*_D_ with different drain voltages *V*_D_ for programming mode (**c**) and erasing mode (**d**).

A critical parameter which decides the device performance is the thickness *d* of the HfO_2_ layer. We calculate time responses of tunneling current densities in programming mode, electrical field strength between drain and graphene, density of stored charge, i.e., *J*_1_, *E*_D_, and *Q*_FG_, in Fig. 4(a) and (b) with *d* as a variable. The drain voltage in the calculation is −5.5 V. It can be seen that reducing the thickness *d* could enhance the electrical *E*_D_ and consequently the amplitude of tunneling current density *J*_1_, which implies a reduced programming time for storing the same amount of electrons in graphene. However, reducing the thickness *d* would deteriorate the retention time, and even the programming process forbidden. As shown in Fig. 4(a), *J*_2_ becomes equal to *J*_1_ at *t* = 0.5 μs for *d* = 3.5 nm. Therefore, a very thin tunneling layer is not advisable. Based on the trade-off between the programming speed and the retention time, a 4.5-nm-thick HfO_2_ is chosen as the tunneling layer.

In Fig. 4(c) and (d), we plot time responses of the device for different amplitudes of programming/erasing signals. Increasing the amplitude of the drain voltage can substantially shorten the pulse duration that is required to store or remove a certain amount of electrons. However, the maximum voltage is limited by the breakdown electrical field of HfO_2_ (17 MV/cm in^42^) and the stability of device. The drain voltages in the programming and erasing modes are finally set as *V*_D_ = −5.5 V and +6 V, respectively. The absolute amplitude of *V*_D_ in the erasing mode is higher and operating time is longer than those in programming mode. This is due to the fact that tunneling electrons experience a higher barrier height in the erasing mode as shown in Fig. 2(l). In order to equalize the programming and erasing times, the pulse amplitudes of the two operation modes should be adjusted accordingly. According to Eq. (1), the leakage current density from graphene to drain is on the order of ~10^−10^ A/cm^2^ provided that the charge density in graphene layer is ~3 × 10^12^/cm^2^. This order of magnitude is in accordance with the calculations in refs^43,44^, and implies a very long retention time in theory. However, since insulating abilities of practical thin dielectric layers cannot be ideal, leakage currents in realistic nonvolatile devices are usually much larger than this theoretical estimation. Here *RC* time constant is not considered in calculation, since it is usually on the order of picoseconds (ps)^5,7^.

### Nonvolatile switching of THz signal

In this part, we study the switching characteristic and the dynamic response of this nonvolatile device. The charges stored in graphene (*Q*_FG_) change the transmission of THz signal by varying its optical conductivity (*σ*_g_(ω)). Since charges can be stored for a long period before an opposite erasing voltage comes, the device hence acts as a nonvolatile THz switch. Optical absorption in graphene includes interband and intraband transitions. However, in THz range the intraband transition dominates, so its optical conductivity can be described by a Drude-like dispersion formula^45^ ${\sigma }_{g}(\omega )=iq{E}_{F}/\pi {\hslash }^{2}(1+{\omega }^{2}{\tau }^{2})$, here τ is the carrier momentum scattering time. The Femi level of graphene is related to the density of stored charges: $|{E}_{F}|=\hslash {\nu }_{F}\sqrt{\pi |{Q}_{FG}|}$, here *ν*_F_ = 10^6^ m/s is the Fermi velocity of Dirac fermions. The transmissivity of THz signal can be calculated as^46^

6 $${\rm{T}}({\rm{\omega }},\,{n}_{s})=\frac{\sqrt{{\varepsilon }_{2}}}{\sqrt{{\varepsilon }_{1}}}\frac{4{({\varepsilon }_{1}{\varepsilon }_{0})}^{2}}{|(\sqrt{{\varepsilon }_{1}{\varepsilon }_{2}}+{\varepsilon }_{1}){\varepsilon }_{0}+\sqrt{{\varepsilon }_{1}}{\rm{Re}}({\sigma }_{g}(\omega ))/c{|}^{2}}$$

here, *ε*_1_ and *ε*_2_ are the permittivities of covering layer and substrate, respectively.

## Discussion

**Figure 5:**
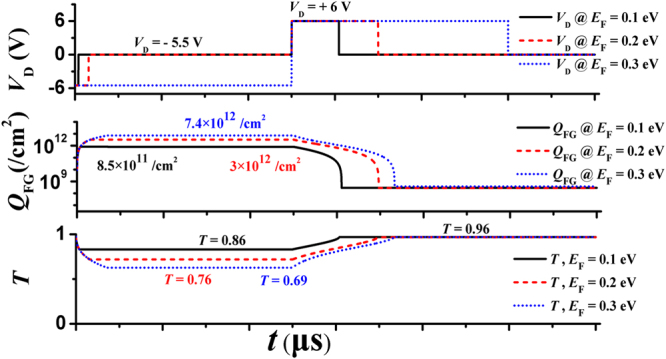
Waveforms of driving signals and the corresponding time responses of the device. *E*_F_ is tuned from 0 eV to 0.1 eV (black, solid), 0 eV to 0.2 eV (red, dash), and 0 eV to 0.3 eV (blue dot) by adding drain voltage pulses of different durations.

According to the analysis above, the amplitudes of the programming/erasing (negative/positive) pulses are chosen to be −5.5 V and +6 V. Waveforms of the programming/erasing pulses applied on the drain and the corresponding responses of the device are shown in Fig. 5. With a 0.4-μs-long programming pulse, electrons in the FG layer finally reach a density of ~8.5 × 10^11^/cm^2^. *E*_F_ of graphene then rises by 0.1 eV. The modulation depth (MD), defined as |*T*(*n*_s_) − *T*(*n*_0_)|/*T*(*n*_0_), is calculated to be 9.5% at 5 THz according to equation (6). The corresponding erasing time is 4.5 μs. If the programming and erasing times are extended to 2.4 μs and 9.6 μs, respectively, more electrons (~3 × 10^12^/cm^2^) are stored, and thus the MD is enhanced to 20%. By further prolonging the programming and erasing time (7.65 μs and 12 μs, respectively), *Q*_FG_ can reach to (~7.4 × 10^12^/cm^2^), meanwhile, its MD is increased to 27%.

**Figure 6:**
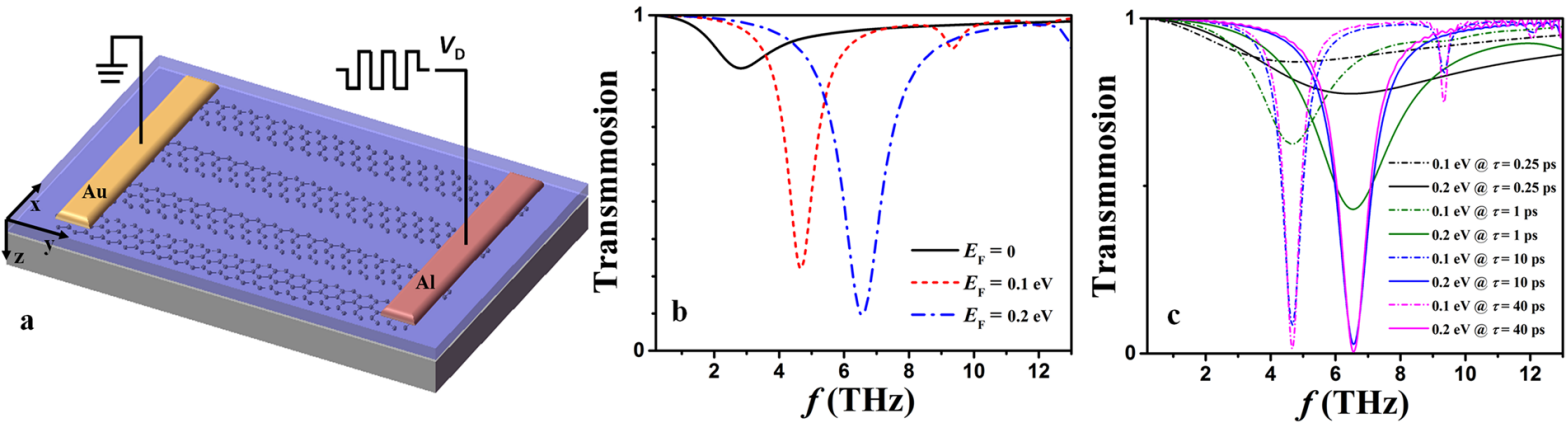
(**a**) Schematic diagram of graphene-ribbon-array based nonvolatile switch. (**b**) Transmission for *E*_F_ = 0 eV, 0.1 eV and 0.2 eV as a function of frequency. (**c**) Transmission for *E*_F_ = 0.1 eV and 0.2 eV as a function of τ.

In accordance with Figs 4 and 5, the MD (or stored charges *Q*_FG_) relies on the duration time of the programming/erasing pulses as long as amplitudes of driving pulses are fixed. Enhancing the MD by using long pulses is in contradiction with improving the operation speed. In order to overcome this tradeoff, we can take advantage of graphene-based plasmons. As collective oscillations of charge carriers, plasmons can occur in patterned graphene^47^. For example, the graphene-ribbon array shown in Fig. 6(a) supports highly confined plasmons which can resonantly couple with the incident light. The response is similar to the monolayer graphene sheet while the incident light polarized along the ribbon, therefore, a normally incident light polarized perpendicular to the ribbons is used to excite the surface Plasmon mode^48–52^. The simulation result shows that this structure helps to enhance the MD remarkably. The modeled resonance curves are presented in Fig. 6(b) by using a three-dimensional finite-different time domain method (Lumerical FDTD Solutions). The simulation condition is given in Methods. Figure 6(b) indicates that the MDs reach 80% (4.6 THz) and 89% (6.5 THz) by tuning *E*_F_ from 0 eV to 0.1 eV and to 0.2 eV, respectively. The power consumption is proportional to the total stored charges and hence the area of the floating-gate layer, the corresponding power consumptions per unit area are 0.0068 J/cm^2^ and 0.0085 J/cm^2^ to tune the Femi level from 0 to 0.1 eV (programming mode) and from 0.1 eV back to 0 eV (erasing mode), respectively. These values become 0.026 J/cm^2^ and 0.033 J/cm^2^ for tuning Femi level from 0 to 0.2 eV and from 0.2 to 0 eV, respectively. The simulation in Fig. 6(b) is based on a scattering time of τ = 3 ps for the graphene on SiO_2_ substrate according to data in^52^. It is observed that both impurities and phonons affect scattering rates of practical graphene layers^53–55^, so the value of scattering time is not fixed but depends on the particular processing condition. To investigate the impact of the scattering time, we also carry out the simulation in Fig. 6(c) with scattering times of 0.25 ps, 1 ps, 10 ps and 40 ps according to the data in^8,51^. It can be seen that a high-quality graphene layer of long scattering time is favorable to realize a narrow resonance curve with high resonance depth, since the conductivity of graphene is proportional to *iτ*^−2^.

In conclusion, we propose a graphene-based nonvolatile THz intensity switch with asymmetric electrodes. If a uniform graphene layer is used as the floating gate layer, the modulation depths are 9.5%/20% by nonvolatile tuning *E*_F_ of graphene between 0 and 0.1/0.2 eV with voltage pulses of appropriate amplitude and duration. Moreover, the MD can be further improved to 89% by patterning the graphene layer into a ribbon array. The performance of this configuration can be further improved by different manners. For example, since charged impurities in oxides introduce disorders of graphene, h-BN can be used to replace HfO_2_ so as to increase the quality of graphene^56^; using multi-layer graphene instead of graphene monolayer is able to enhance the effective refraction index variation^19^, and so on.

## Methods

The numerical simulation of electrostatic potential distribution and the corresponding electrical distribution as shown in Fig. 1 is performed by the electrostatic module of commercial software (Comsol Multiphysics) based on finite element method (FEM). The free-triangular mesh is employed and the electrical properties of graphene are derived from refs^8,9^. The width of electrodes (drain and source) is *W*_1_ = 1 mm; and that of the floating-gate layer is *W*_2_ = 1 cm. Since *W*_1_ and *W*_2_ are several orders of magnitude larger than the thickness of graphene, they are scaled down to 5 nm and 50 nm in the simulation for a better eye-capture.

The tunneling processes of electrons in programing/erasing modes are calculated by Matlab based on the model in refs^31,34–41^; the results are shown in Figs 3 and 4. The effective drain voltage *V*_D_eff_ is calculated via the equations in refs^39–41^. The voltage at the Dirac point is normally related to work function difference between metal and graphene as well as interface trap states. In this study we assume the graphene is ideal, so only the effect of different work functions is considered. The transmissivities through the monolayer graphene in Fig. 5 are also calculated by Matlab based on equation (6). Three chemical potentials (Femi levels) are considered, i.e., 0, 0.1, 0.2 eV, in Fig. 5.

The numerical simulation of the THz transmissivity through graphene ribbons is performed with the 3-D finite-different time domain method (Lumerical FDTD Solutions). The result is shown in Fig. 6. The graphene ribbons are oriented along the *y* direction. A plane-wave source is normally incident (*z*-direction) on graphene. The periodic boundary condition is used along the *y* direction, while the anti-symmetric boundary condition is used along the *x* direction. The perfectly matched layer (PML) is set at the *z*-direction. The graphene material type (temperature of 300 K) together with the 2D rectangle geometry described in modeling methodology are employed to model the graphene layer following the surface conductivity approach. The grid size for graphene layer is Δ*x* = Δ*y* = 2.5 nm and Δ*z* = 0.25 nm.

## Electronic supplementary material

Supplementary Information
