# Supplementary material for: Graphene-based nonvolatile terahertz switch with asymmetric electrodes
Source: Sci Rep. 2018 Jan 24;8:1562. doi: 10.1038/s41598-018-20047-3 (PMC5784144; doi:10.1038/s41598-018-20047-3)
Supplement: Supplementary file 1 — Supplementary Information [file 41598_2018_20047_MOESM1_ESM.pdf]

Supplementary Information

Graphene-based nonvolatile terahertz switch with asymmetric electrodes

Yan Li[1,2], Hui Yu[1*], Xinyu Qiu[1], Tingge Dai[1], Jianfei Jiang[1], Gencheng Wang[1], Qiang Zhang[1], Yali Qin[2], Jianyi Yang[1], and Xiaoqing Jiang[1]

[1]College of Information Science and Electronics Engineering, Zhejiang University, Hangzhou 310027, China.

[2]College of Information Engineering, Zhejiang University of Technology, Hangzhou 310014, China.

[*]Correspondence and requests for materials should be addressed to Z.J.U. (email: huiyu@zju.edu.cn)

Tunneling current density in Metal/oxide/graphene and Metal/oxide/Metal system

Assuming a barrier is in the x direction, the transmission probability D that an electron can penetrate the barrier depends only on the energy component E_x of the incident electron in the x direction. It can be calculated by the well-known WBK approximation as $D = \exp(-2K)$, where

$$K = \int_{x_1}^{x_2} \kappa(x, E_x)\mathrm{d}x, \tag{S1}$$

$$\kappa(x, E_x) = \{\frac{8\pi m^*[V(x)-E_x]}{h^2}\}^{1/2} \tag{S2}$$

Here, parameters x_1 and x_2 denote limits of the barrier at Femi level, m^* is the effective mass of electrons, while $V(x)$ is the height of the potential barrier. With the definition of the transmission probability, the tunneling current density then can be described as [S1]

$$J = \frac{q}{2\pi^2 h}\int_0^{\infty} \mathrm{d}E[f(E) - f(E+eV)] \iint \mathrm{d}^2 k_t D(E) \tag{S3}$$

If the thickness of the electrode is extremely small, e.g., the one-atom-thick graphene layer, the density of states for the two-dimensional motion of electrons would affect the tunneling. The expression of tunneling current density in Eq. (S3) can be reorganized as

$$J = \frac{2q}{h}\int_{-\infty}^{\infty} \mathrm{d}E[f(E) - f(E+eV)] \int_0^E \rho(E_t) D(E_t)\mathrm{d}E_t \tag{S4}$$

where E_t and ρ are the energy component and the density of states for the two-dimensional motion of electrons in the transverse direction, respectively.

The electronic energy dispersion of graphene is $E_t(p) = \pm\frac{hv_0}{2\pi}\sqrt{(k_z^2 + k_y^2)}$. The corresponding density of states can be expressed as [S3]

$$\rho(E_t) = \frac{\mathrm{d}N}{\mathrm{d}E_t} = \frac{2\pi g_s g_v |E_t|}{(hv_F)^2} \tag{S5}$$

where g_s and g_v are the spin and valley degeneracies, both of which are 2.

By substituting Eq. (S5) into Eq. (S4), we obtain the tunneling current density in a metal-oxide-graphene junction

$$J_g = \frac{2q}{h}\int_{-\infty}^{\infty} \mathrm{d}E[f(E) - f(E+eV)]\int_0^E \frac{2\pi g_s g_v |E_t|}{(hv_F)^2} D(E_t)\mathrm{d}E_t$$
$$= \frac{8\pi q g_s g_v}{A^4 h^3 (v_F)^2}\int_{-\infty}^{\infty} [f(E) - f(E+eV)][(A^3(\eta - E)^{3/2} + 3A^2(\eta - E) + 6A(\eta - E)^{1/2} + 6)e^{-A(\eta - E)^{1/2}}] \quad \text{(S6)}$$

where $A = 4\pi\beta t\sqrt{2m^*}/h$, t is the thickness of the tunneling-oxide layer and $\beta \simeq 1$ [S3]. In comparison, the conventional Fowler-Nordheim (FN) formula used in the main text is expressed as

$$J = \frac{q^3}{8\pi h \emptyset_B} E^2 \exp\left(\frac{-8\pi\sqrt{2m^*}(\emptyset_B - E_F)^{3/2}}{3hqE}\right) \quad \text{(S7)}$$

We compare calculation results based on equations (6) and (7) at different Femi levels E_F of graphene. For example, $J_g/J \approx 0.15$ and 0.43 at $E_F = 0.2$ eV and 0.4 eV, respectively. Although the conventional FN model doesn't take the peculiar band structure and density of states of graphene into account, it produces a result of acceptable accuracy. However, the benefit of using the conventional FN model is that the close-form expression of tunneling current density in Eq. (S7) greatly simplifies the calculation.

Supplementary References:

S1. E. L. Wolf, Principles of electron tunneling spectroscopy. Oxford University Press (1985).

S2. Sanchez-Yamagishi, J. et al. Scanning tunneling microscopy and spectroscopy of ultra-flat graphene on hexagonal boron nitride. Nat. Mater. **4**, 282-285 (2011).

S3. Simmons, J. G. Generalized formula for the electric tunnel effect between similar electrodes separated by a thin insulating film. J. Appl. Phys. 34, 1793-1803 (1963).
